# Pulse noise-hidden image reconstruction and visualization via stochastic resonance

**DOI:** 10.1038/srep36678

**Published:** 2016-11-08

**Authors:** Qibing Sun, Hongjun Liu, Nan Huang, Zhaolu Wang, Jing Han

**Affiliations:** 1 State Key Laboratory of Transient Optics and Photonics, Xi’an Institute of Optics and Precision Mechanics of CAS, Xi’an, 710119, China

## Abstract

We investigate the nanosecond pulse noise-hidden image reconstruction and visualization using stochastic resonance implemented by modulation instability. In particular, this dynamical stochastic resonance holds with coupling between the pulse incoherent noise and pulse coherent signal, and provides a substantial enhancement of the signal-to-noise ratio and cross-correlation. This means that the pulse noise-hidden image can be effectively reconstructed with high visibility and fidelity via stochastic resonance at appropriate system parameters. Such a simple and convenient method has potential applications in image processing under noisy environment.

Weak optical signal processing and detection is a novel and rapidly developing research direction that aims to effectively extract and reconstruct the wanted signals from the strong noise background. It has very important applications in laser radar, laser sensing, biomedicine, laser imaging and many other low-level signal detection fields. However, the measured signal may be suppressed under the noise elimination process that appears in the traditional detection methods. Noise is usually considered detrimental to the signal restoration process and will degrade the performance of dynamical systems, which leads to great difficulty in low-level signal detection, especially for the noise-hidden signals. This promotes the development of an alternative idea, that is, whether the harmful noise can become beneficial for the low-level signal processing and detection. In some special nonlinear systems, the presence of noise can improve the signal-to-noise ratio (SNR) and visibility of the weak signals, exhibiting the phenomenon of stochastic resonance^1^. Compared with the conventional linear systems, this unique characteristic offers a great potential for noise-hidden signal reconstruction with lower SNR and appears in a variety of physical systems^2^,^3^,^4^,^5^. The classic system for stochastic resonance is induced by a particle oscillating periodically in a double-well potential and usually restricted to a threshold^2^,^3^,^4^, while there is no need for feedback or threshold in the new type of stochastic resonance based on the modulation instability^5^, making it more simple and flexible in the practical applications. This means that it is appropriate for nonlinear filtering of the weak signals and usually focuses on two-dimensional images rather than one-dimensional signals. D. V. Dylov *et al*. have exploited this stochastic resonance using continuous wave in a self-focusing medium^5^, which suggests a general method for reconstructing images through seeded instability. At present, with the rapid development of the laser radar and other imaging fields, the pulse image has extensive and important applications in many optical signal processing fields^6^,^7^,^8^. After transmission in the complex environment, the measured optical signal is usually low-level and submerged by the high noisy background. Once this signal is processed and detected with the introduction of such a nonlinear element into the optical path, the conventional techniques can also be used to improve its quality further. Jing Han *et al*. have demonstrated the reconstruction of pulse noisy images via stochastic resonance in theory^9^, providing reference for the experimental investigation. However, the main criticism of a novel technology mainly concerns the experimental validation. To further prove the feasibility and effectiveness of pulse noise-hidden image reconstruction and visualization via stochastic resonance, it is essential to experimentally clarify the influences of the nonlinearity tuned by the applied electric field, signal-to-noise intensity ratio and repetition rate of the pulse image on the noise-hidden signal reconstruction via stochastic resonance. On this basis, it is also need to experimentally specify how to obtain the optimal output by tuning the system parameters. This will promote the progress of stochastic resonance and its applications in many optical imaging fields.

In this paper, we report the reconstruction of nanosecond pulse noise-hidden images via stochastic resonance, displaying high SNR enhancement and cross-correlation gain. The nanosecond noise-hidden images grow at the expense of the pulse noise and become visible by optimizing the system parameters, leading to the detection sensitivity improvement of the weak optical signals. As with the continuous signal and noise, a significant feature is that the visibility of the pulse noisy images varies within the pulse duration. These conclusions will further improve and perfect the stochastic resonance system, which accelerates its practical applications for weak optical image processing and detection under high noisy environment.

## Results

### Experimental setup

**Figure 1:**
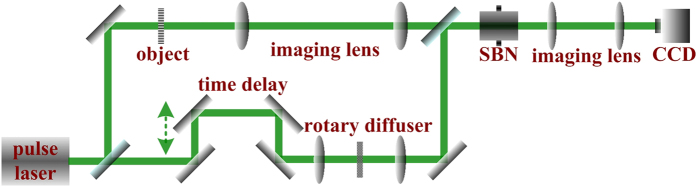


As with the previously reported designs^5^, a pulse incoherent noise and a pulse coherent signal are employed to explain the nature of pulse noise-hidden image processing based on stochastic resonance. In addition, the nonlinear system is the last key elementary component determining the output performance of this proposed method. Figure 1 presents the detailed schematic of this stochastic resonance produced by modulation instability. The pulse signal is purely coherent and an image of a resolution chart formed by the 4-f optical system. The pulse noise is spatially incoherent with random phase fluctuations and generated by a “lens-diffuser-lens” system. The signal and noise pulses are divided from a same nanosecond pulse laser with the wavelength of 532 nm and pulse width of about 17 ns, in which a time delay line is used to guarantee the synchronization in time domain. The nonlinear medium is a 5.5 × 5 × 10 mm^3^ SBN:75 photorefractive crystal doped with CeO_2_, whose strength of the photorefractive nonlinearity can be controlled by the applied electric field parallel to its optical axis. The pulse signal and pulse noise are spatially overlapped and collinearly injected into the SBN:75 crystal. Nonlinear interaction between the signal and noise will take place during the nonlinear stochastic resonance process and light exiting the photorefractive crystal is then imaged by a 4-f optical system onto a CCD camera.

### Numerical results and analysis

To better understand and exploit the stochastic resonance with a pulse signal and a pulse noise, the instability-linear perturbation theory is utilized to numerically analyze the dynamic coupling process, in which the perturbation treats each pixel individually for the low-level pulse images^9^,^10^,^11^,^12^. To make the theory analytically tractable, the coherent mode-mixing within the signal is ignored and the noise is treated as a drive term acting on the signal. That is, we focus on the response of the pulse noise to the driven pulse signal that provides valuable insight into mechanism for the coherent-incoherent coupling^9^,^10^,^11^,^12^. This allows a more proper treatment for the incoherent dynamics. Considering the low-level intensity of the injected light and high transmittance of the nonlinear medium, the linear loss and high-order nonlinear contribution are neglected and thus the growth rate *g* can be expressed as^2^,^9^


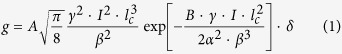


where *A* and *B* are effective mode-dependent normalization constants giving the height and location of the visibility peak, *γ* is the nonlinear coefficient of spatial coupling electric field, *I* is the incident light intensity, *β* = *λ*/2*πn*_*0*_ is the diffraction coefficient for light of wavelength *λ* in a medium with index of refraction *n*_*0*_, *l*_*c*_ is the correlation length, *α* is the wavenumber, *δ* indicates the Gaussian distribution in the time domain of nanosecond pulse, respectively.

**Figure 2:**
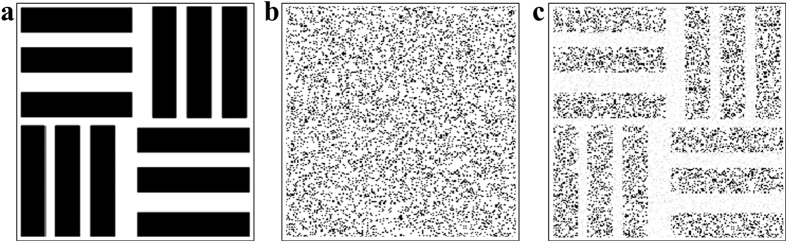
Output performance of stochastic resonance. (**a**) original image, (**b**) noise-hidden image and (**c**) output image.

To evaluate and manifest the low-level pulse signal processing ability under the high noisy background via stochastic resonance, the initial signal-to-noise intensity ratio is fixed at 1:45 with the average signal power of 100 nW. That is, the input image is highly noise-hidden and invisible. The numerical simulations of this nonlinear stochastic resonance system are performed by solving Eq. (1). The parameters are defined as *A* = 1.6, *B* = 530, *n*_*0*_ = 2.3 and *l*_*c*_ = 100 μm 5. At a fixed correlation length of the pulse noise, the optimal output image is obtained by adjusting the photorefractive nonlinearity, as presented in Fig. 2. It is clear that the visibility is greatly improved due to the instability of energy coupling between the coherent signal and random noise as the nonlinear change of the refractive index Δ*n* keeps acting on the interface. This leads to the reconstruction of the distribution of total energy between beams with different coherence through the movement of particles. As a consequence, the SNR is significantly enhanced resulting in the visualization of the noise-hidden image, where nonlinearity allows the signal reconstruction and visualization through the modulation instability.

**Figure 3:**
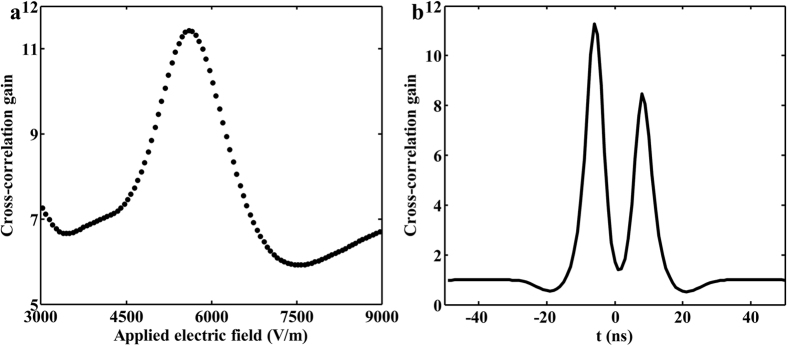
Cross-correlation gain varying (**a**) with the applied electric field and (**b**) within the pulse duration.

To estimate the performance of this pulse stochastic resonance system, the cross-correlation gain is used as a quantitative measure of the quality improvement between the input image and the output image, which is defined as reference^13^,^14^. Figure 3(a) illustrates the relationship between the cross-correlation gain and the applied electric field *E*. At a fixed *E*, the maximum cross-correlation gain within the pulse duration is evaluated. It is obvious that the cross-correlation gain varies with the applied electric field, where exists an optimal value for acquiring the maximum cross-correlation gain. As seen from Eq. (1), the output image is mainly determined by the nonlinearity, intensity and correlation length of the noise. The change of Δ*n* and photorefractive nonlinearity varies with the applied electric field, which determines the energy transferring efficiency from high-level noise to low-level signal. In addition, compared with the continuous stochastic resonance, the cross-correlation gain is changing within the pulse duration in the pulse stochastic resonance as shown in Fig. 3(b). This is mainly induced by the various intensities at different time points of the input pulse. The spatial coupling electric field in the photorefractive crystal is established with a form of oscillation^15^. The nonlinear coefficient *γ* is dynamical because of the fluctuant spatial coupling electric field, which leads to a different growth rate *g* and output image. Namely, the quality and visibility of output images mainly depends on the applied electric field, intensity and correlation length of the noise.

### Experimental results and analysis

**Figure 4:**
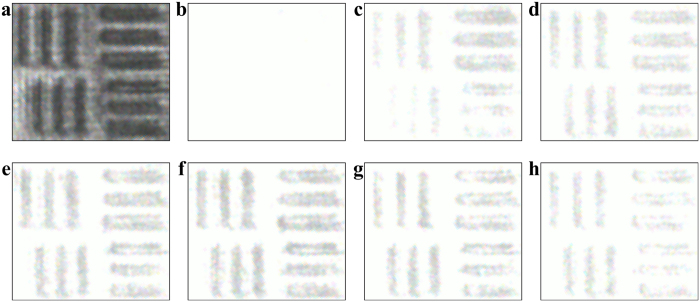
Pulse noise-hidden image reconstruction at different applied voltages. (**a**) Pure image, (**b**) noise-hidden image, (**c**) 150 V, (**d**) 200 V, (**e**) 250 V, (**f**) 300 V, (**g**) 350 V and (**h**) 400 V.

**Figure 5:**
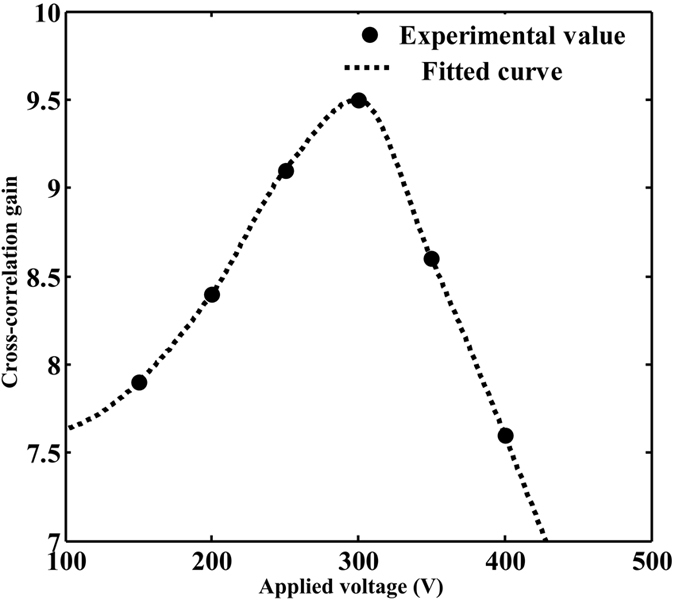


The experiment for the noise-hidden pulse image reconstruction and visualization was carried out using the scheme displayed in Fig. 1. To improve the practicability and compatibility of stochastic resonance, the parameter-tuning method is proposed and employed for acquiring the optimal output visibility. The spatial correlation length of the pulse noise is fixed under an initial signal-to-noise intensity ratio, which is depended on the incident spot size on the diffuser and its rotation speed^16^. As a consequence, the output performance of this dynamical stochastic resonance is optimized by carefully adjusting the applied voltage across the crystal that determines the nonlinear change of the refractive index Δ*n* of the photorefractive crystal^2^,^9^. At an initial signal-to-noise intensity ratio of about 1:45, the optimal visibility of the output images at different applied voltages is recorded by the CCD and shown in Fig. 4. It can be seen that the visibility varies with the applied voltage and the pulse noise-hidden image becomes more and more clear as the applied voltage increases to about 300 V. That is, a positive exchange is established during the stochastic resonance process based on the modulation instability, leading to the reconstruction of noise-hidden images. This is due to the variation of the nonlinear change of the refractive index Δ*n* and nonlinear coefficient of the photorefractive crystal that can be adjusted by the applied voltage. Specially, the very weak signal first seeds a potential that concentrates the noise, in turn, nonlinear coupling amplifies the signal and reinforces the potential. Namely, the initial signal is essentially a seed that triggers an instability in the noise. Through this seeded instability, the pulse noise-hidden signal will be reconstructed and visualized with high fidelity, where the intensity perturbations grow at the expense of a uniform background. However, the output image becomes blurred again as the applied voltage increases further, as seen in Fig. 4. At a higher Δ*n*, the turbulent dynamics will continue mixing the pulse signal and noise, whose border degrades in coherent and incoherent snake-like instability^17^,^18^. This indicates that too much nonlinearity will degrade the resonance pattern and thus appropriate nonlinearity is necessary for the pulse noise-hidden image reconstruction and visualization. While variation of the visibility of the output image within the pulse duration is not observed due to the lager exposure time of the CCD. To evaluate the ability of pulse noise-hidden image processing, the cross-correlation gain is also analyzed and found to be in close agreement with the theoretical results. As depicted in Fig. 5, the cross-correlation gain varies with the applied voltage and a maximum value of about 10 is obtained at the applied voltage of 300 V. These results show that parameter-tuning is vital for the optimization of stochastic resonance, which provide important reference for adaptive stochastic resonance.

**Figure 6:**
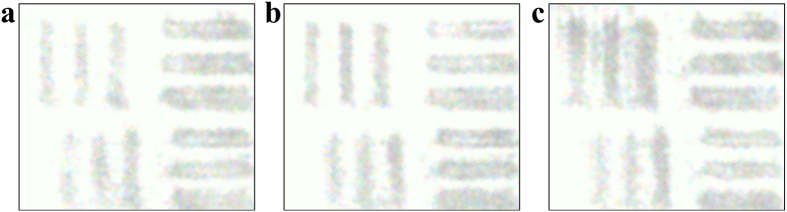
Optimal output images at different noise intensities. Initial signal to noise intensity ratios are (**a**) 1:50, (**b**) 1:55 and (**c**) 1:60.

The generalized parameter-tuning stochastic resonance is introduced based on the tuning of applied voltage, which has incomparable flexibility and is highly effective in practical applications of image processing. The initial signal-to-noise intensity ratios are set as 1:50, 1:55 and 1:60. The applied voltages are carefully adjusted to acquire better visibility, as illustrated in Fig. 6. It is clearly seen that the output pulse images become visible with the cross-correlation higher than 8 under the voltages of 300 V, 350 V, and 400 V. That is, the modulations become more pronounced with higher visibility by optimizing the applied voltage when the initial signal-to-noise intensity ratio is fixed. This typical characteristic is induced by the instability of energy coupling between weak signals and random noise related to the nonlinearity^2^,^9^. However, too much noise will dominate this system and destroy the conditions of stochastic resonance at a fixed applied electric field, which leads to the distortion of output images. In a word, all the above results strongly indicate that the pulse-noise hidden images can be effectively reconstructed and visualized via parameter-tuning stochastic resonance based on modulation instability.

**Figure 7:**
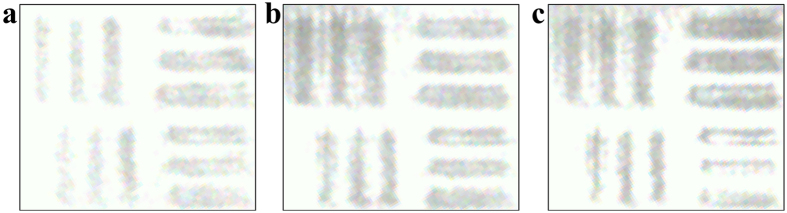
Output images at different repetition frequencies. (**a**) 400 Hz, (**b**) 700 Hz and (**c**) 1 kHz.

In many imaging fields, the repetition frequency of the pulse signals is different according to the practical application acquirements. At a fixed signal-to-noise intensity ratio of 1:45 and applied voltage of 300 V, the noise-hidden image processing via stochastic resonance at different repetition frequencies is presented in Fig. 7, in which the exposure time of CCD is set to guarantee about 10 pulses to build the output images. It is clearly seen that the noise-hidden images are effectively extracted with a high visibility, when the repetition frequencies are 400 Hz, 700 Hz and 1 kHz, respectively. Moreover, the cross-correlation gains are all greater than 8 at various repetition frequencies. That is, the pulse noise-hidden image reconstruction and visualization via stochastic resonance can allow a large repetition frequency range, which expands the application fields of stochastic resonance.

## Discussion

The stochastic resonance based on modulation instability is a very flexible and general approach for pulse noisy image processing. It holds with coherent-incoherent coupling for certain system parameter ranges. The nonlinear coupling is necessary to cause the energy transfer between different components, in which diffraction and nonlinearity determine the intensity of incident wave enhanced or diminished in propagation^19^,^20^. This promotes the development of parameter-tuning stochastic resonance, where tuning parameters suitably can make the energy transfer from the strong noise to the relatively weak signal. Under appropriate system parameters, the pulse noise-hidden images grow at the expense of noise and become visible with a peak cross-correlation gain, which can be used for signal reconstruction and contrast enhancement. These characteristics reflect the phenomena of stochastic resonance that is compatible with the existing imaging systems. This paves the way for a variety of applications of stochastic resonance and provides an effective means to improve the detection sensitivity in weak signal processing and detection fields.

In conclusion, we have presented an innovative and practical technology for pulse noise-hidden image reconstruction and visualization based on stochastic resonance. It can effectively reconstruct nanosecond noise-hidden images with a cross-correlation gain of about 10 by carefully adjusting the system parameters, which extends the applications of stochastic resonance. The experimental results agree well with the predictions from numerical simulations. This provides an efficient method for processing and detecting pulse noise-hidden images in various imaging fields.

## Additional Information

**How to cite this article**: Sun, Q. *et al*. Pulse noise-hidden image reconstruction and visualization via stochastic resonance. *Sci. Rep.* **6**, 36678; doi: 10.1038/srep36678 (2016).
